# Adaptive feature extraction method for capsule endoscopy images

**DOI:** 10.1186/s42492-023-00151-6

**Published:** 2023-12-11

**Authors:** Dingchang Wu, Yinghui Wang, Haomiao Ma, Lingyu Ai, Jinlong Yang, Shaojie Zhang, Wei Li

**Affiliations:** 1 School of Artificial Intelligence and Computer Science, Jiangnan University, Wuxi, 214122 Jiangsu China; 2 School of Computer Science, Shaanxi Normal University, Xian, 710062 Shaanxi China; 3 School of Internet of Things Engineering, Jiangnan University, Wuxi, 214122 Jiangsu China

**Keywords:** Capsule endoscopy, Feature extraction, Adaptive threshold

## Abstract

The traditional feature-extraction method of oriented FAST and rotated BRIEF (ORB) detects image features based on a fixed threshold; however, ORB descriptors do not distinguish features well in capsule endoscopy images. Therefore, a new feature detector that uses a new method for setting thresholds, called the adaptive threshold FAST and FREAK in capsule endoscopy images (AFFCEI), is proposed. This method, first constructs an image pyramid and then calculates the thresholds of pixels based on the gray value contrast of all pixels in the local neighborhood of the image, to achieve adaptive image feature extraction in each layer of the pyramid. Subsequently, the features are expressed by the FREAK descriptor, which can enhance the discrimination of the features extracted from the stomach image. Finally, a refined matching is obtained by applying the grid-based motion statistics algorithm to the result of Hamming distance, whereby mismatches are rejected using the RANSAC algorithm. Compared with the ASIFT method, which previously had the best performance, the average running time of AFFCEI was 4/5 that of ASIFT, and the average matching score improved by 5% when tracking features in a moving capsule endoscope.

## Introduction

### Background

Regular classical feature-extraction methods, such as scale-invariant feature transform (SIFT) [1], speed-up robust features (SURF) [2], features from accelerated segment test (FAST) [3], and oriented FAST and rotated BRIEF (ORB) [4] are widely used in realistic scenarios.

Although these popular methods have achieved good results in indoor and outdoor scenes [5–7] and can extract sufficient features for wired endoscopic images from bladder [8] and viscera [9, 10], they are not the best for capsule endoscopic scenes compared to other environments. The underlying reason for this is the presence of low texture, specular reflection, and high light intensity in endoscopic images. The combined interference of these joint factors makes effective extraction of features difficult, complicating the completion of later tasks such as feature matching, positional estimation, and 3D point calculation for capsule endoscopic images. To ensure that the feature-extraction method is effective for capsule endoscopic images, researchers have used artificial enhancement strategies. For example, some researchers have used projection devices to add structured light patterns to surfaces with less texture [11]. Others have sprayed the indigo carmine (IC) dye onto the stomach to improve imaging conditions [12–14]. These techniques aim to improve the environment and facilitate accurate feature extraction.

The aforementioned methods entail the use of additional equipment and more significantly, may adversely affect or even harm the patient. Moreover, physicians are generally reluctant to introduce additional procedures for lesion diagnosis. Therefore, these techniques may not be feasible in specific clinical settings. To address these issues, this study proposes an adaptive feature-extraction method for capsule endoscopy images, called adaptive threshold FAST and FREAK in capsule endoscopy image (AFFCEI), which draws inspiration from the ORB method. This technique aims to enhance feature extraction by adapting to the specific imaging conditions of the capsule endoscope, without introducing additional equipment or potentially harmful procedures. The method first constructs an adaptive threshold based on FAST to achieve the localization of features and then combines the variability of visual sensitivity to feature distance with proximity to realize feature description based on the FREAK [15] descriptor.

The innovative contributions of this study are summarized as follows:

1. An adaptive threshold feature-extraction method is proposed to solve the problem of insufficient FAST feature extraction.
2. The FREAK descriptor is combined with the feature-extraction method proposed in this study to enhance feature distinction in capsule endoscopy images.

### Related work

Feature extraction methods are widely used in many fields, such as target detection and image tracking.

The SIFT algorithm which was proposed in 2004, displays strong robustness to image scale changes and has been a milestone for the feature extraction task. Many improved methods [16, 17] are based on SIFT which simplifies the process of feature extraction and provides satisfactory results not only for natural scenes but also for handheld endoscopic images [18, 19]. However, SIFT and related improved methods are not well adapted to feature extraction for capsule endoscopic images because of the elimination of the edge response and for lacking speed when extracting features.

To address this issue, for faster feature extraction, Rosten proposed the FAST corner detection algorithm [3], which compares the value of the central pixel with the surrounding 16 pixels, and if the absolute difference between the value of 12 consecutive pixels and the value of the central pixel is greater than a preset threshold, the central point is a FAST corner. Because FAST corners are not scale- or rotation-invariant, Rublee proposed the ORB algorithm [4], which is popular among researchers because it reduces the complexity of the FAST algorithm while ensuring the accuracy of feature extraction [20]. ORB uses FAST corner detection on the scale space and the rotation-aware BRIEF (rBRIEF) [21] descriptor to describe features, imparting rotational invariance to the features. Experiments have also shown that the ORB algorithm can achieve good results for organs, such as liver images captured by endoscopy [22]. However, ORB does not perform well on the stomach in capsule endoscopy because of low texture, specular reflection, and high light intensity of capsule endoscopic images. The rBRIEF descriptor used in the ORB algorithm is constructed using a greedy exhaustive method to obtain random point pairs with low correlation. Although this method is faster, it reduces accuracy and cannot distinguish features as well. ORB uses the FAST method for feature extraction, essentially comparing the grayscale values of the pixel points. The fixed threshold value used in the FAST method also leads to instability in feature extraction. Despite these limitations, the ORB algorithm has demonstrated unique advantages and is a popular choice for image feature extraction in both extra-cavity scenes and internal cavity environments, outperforming many classical feature extraction algorithms [22].

Spyrou and Iakovidis [23] compared and evaluated the performance of feature-extraction methods in capsule endoscopy. The ORB method exhibited significant advantages over the other algorithms. Subsequent research improved the selection of thresholds. In ORB_SLAM2, Mur-Arta and Tardós [24] introduced a dual-threshold approach to optimize feature extraction. However, this algorithm sets thresholds artificially without considering pixels. Ma et al. [25] used a dynamic local threshold instead of a fixed threshold, which allows the extraction of additional features through local thresholds calculated based on neighborhood image blocks. However, this method only considers average grayscale values, leading to poorer performance on capsule endoscopy images.

With the development of deep learning (DL), DL concepts have also been applied in feature extraction methods [26–29]. SuperPoint [30] is a representative algorithm for these methods. This method first crops an image to obtain a feature map using VGGnet, where a feature extractor is used to output the probabilities of pixels as features in the feature map. The positions of the features are then determined using non-maximum suppression. In the feature description stage, the feature map dimensions are first expanded, and descriptors are obtained by linearly interpolating the results of feature extraction. This method has achieved favorable results in natural scenes; however, the performance of SuperPoint largely relies on training data and pretrained models. Training data that are not sufficiently diverse and representative may limit the generalizability of the model, leading to performance degradation in certain situations. Additionally, the performance of this method has not been validated in capsule endoscopy environments. Notably, these methods lack strong interpretability.

This study focuses on the threshold variability strategy, and inspired by ORB, proposes an adaptive feature extraction method, AFFCEI, for capsule endoscopy. The core idea of the method is to dynamically determine the threshold value based on a pixel point and remaining grayscale values in a specific neighborhood using the formula proposed in this study, thereby achieving a self-adaptive threshold value, which combined with the FREAK descriptor [15], can better distinguish stomach features as well as describe the extracted features. It is also important to note that the FREAK method provides directional information when constructing the descriptor and maintains the same feature direction invariance as the ORB method.

## Methods

**Fig. 1:**
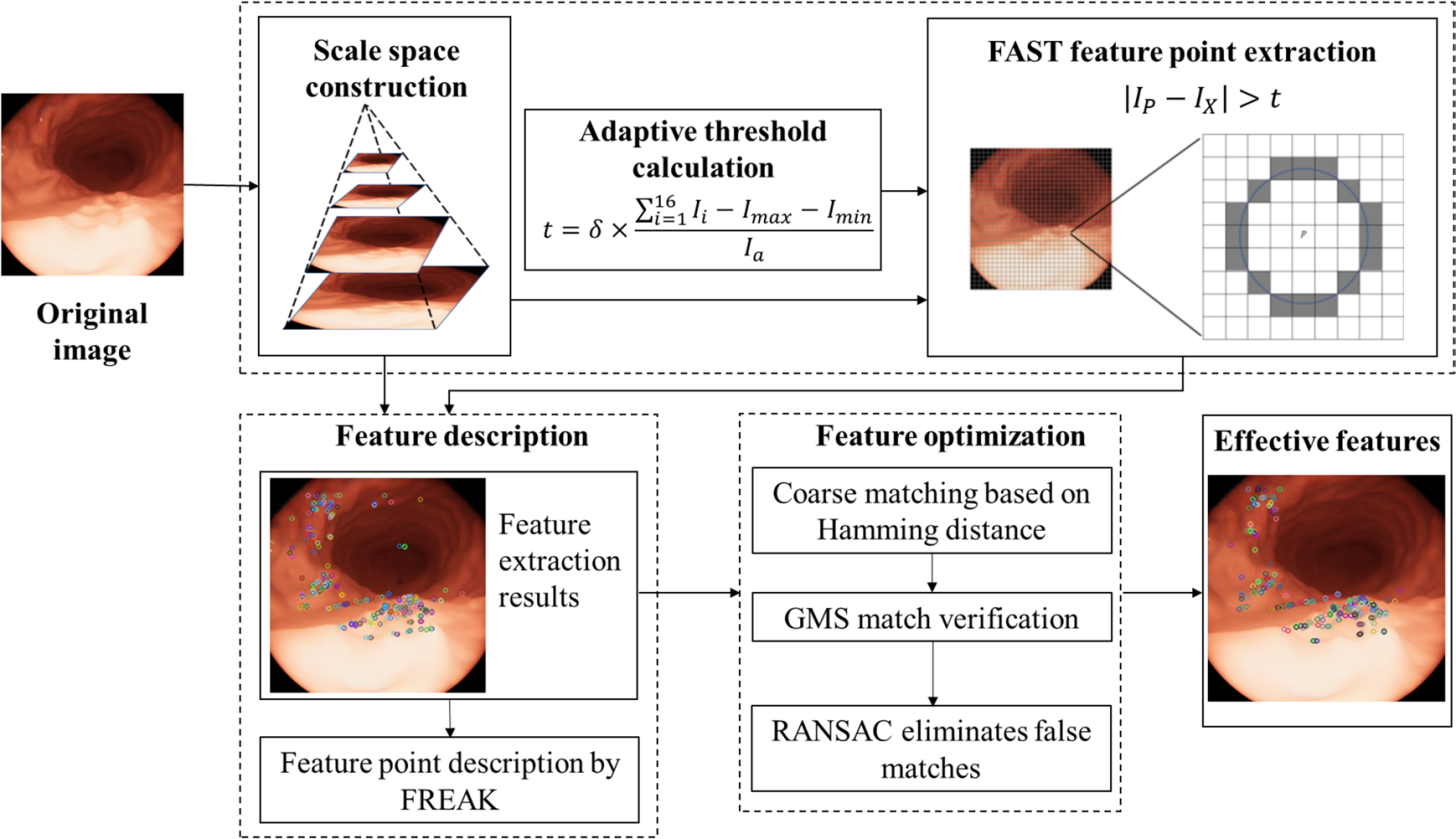
Framework of proposed method

The methodological approach of this study is illustrated in Fig. 1. First, a scale space is constructed to impart feature-scale invariance [1]. Second, a variable threshold calculation method is designed for FAST to implement an adaptive threshold. Third, a feature descriptor based on FREAK is designed to enhance the effective discrimination of gastric features based on sensitivity differences of the features with respect to simulated human eye visual distances. Finally, in the experiment, a two-level feature matching (coarse and fine) is implemented based on the Hamming distance and grid-based motion statistics (GMS) [31] algorithm, effectively eliminating misaligned point pairs through random sample consensus (RANSAC).

### Scale space construction

To ensure the scale invariance of the extracted features, a Gaussian pyramid is first constructed. The original image is used as the first level, and a Gaussian filter is applied to the original image to simulate scale changes and reduce resolution. The image is then downsampled by a scale factor of 1.5 to obtain the second-level image. The same process is repeated for the new image to obtain eight images. Feature extraction is performed on each of the resulting images, recording the layer number where each feature is located.

### Adaptive threshold calculation

When extracting features, a threshold value *t* must be set to determine whether a pixel is a feature. In traditional methods, this threshold value is usually set manually to a fixed value based on experimental results and is applied to all images at different scales. In capsule endoscopy, the camera light source affects the image, with higher brightness in the near field and darker brightness in the far field. As the capsule endoscope moves, the same scene can be affected by different lighting conditions, and applying a fixed global threshold to all pixels is difficult. Therefore, an adaptive threshold is required to increase the accuracy of feature extraction and obtain a sufficient number of features. In this study, the threshold value is associated with the local contrast of the image: 16 pixel points on a circle with the pixel point as the center and three neighboring pixel points with the neighborhood determined by the selected radius [16]. To avoid the influence of noise points and lighting anomalies on the reasonableness of the threshold, the threshold value *t* was calculated as shown in Eq. (1).

1 $$\begin{aligned} t=\delta \times \frac{\sum _{i=1}^{16} I_{i}-I_{\max }-I_{\min }}{I_{a}} \end{aligned}$$

where $\delta$ is a global fixed parameter; $I_{\max }$ and $I_{\min }$ represent the maximum and minimum values in the neighborhood, respectively; and $I_{a}$ is the average of 16 pixel gray scales in the neighborhood. The essence of the FAST algorithm is the measurement of the contrast of adjacent pixels. The pixel that is commonly referred to as the center point, is not the same as the point on the circumference of the circle. Therefore, in this study, the threshold value is set to be proportional to the local contrast of the image. This method subtracts the maximum and minimum values, thereby preventing the effects of extreme values, such as the influence of noise points and lighting anomalies. To prevent the simultaneous elimination of normal points, the denominator is set to the average of 16 pixel gray scales.

After acquiring the images by capsule endoscopy, a Gaussian pyramid is constructed from the images, and features are extracted for each layer of the pyramid individually. When feature extraction is performed on images acquired in spaces of different scales, each pixel of the image has a different threshold value. In particular, in the case of uneven illumination, which is the main influencing factor, the adaptive threshold can adapt well to the variation in each pixel.

### Feature extraction and description

**Fig. 2:**
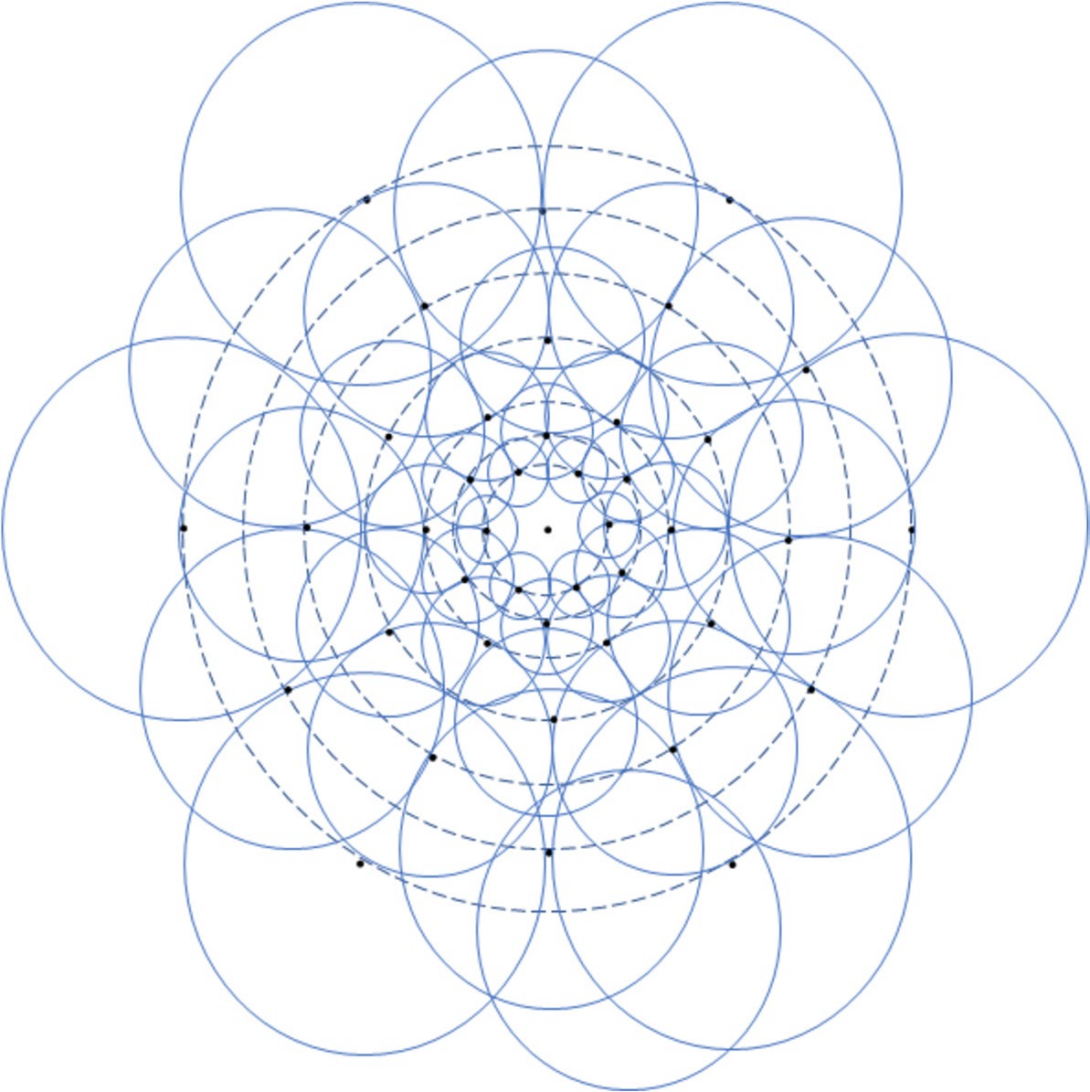
Sampling mode of FREAK

The proposed method for feature extraction is based on FAST [3] with the threshold value calculated using the adaptive threshold Eq. (1) proposed in this study. After extraction, the features are described using the FREAK descriptor [1]. This binary descriptor imitates the human retinal visual mechanism in describing features, sampling images, similar to the human retina. FREAK constructs seven concentric circles with the feature at the center and samples around it. Six sampling points are obtained on each concentric circle, spaced evenly at $60^{\circ }$intervals. The perceptual field is then created with the sampling point at its center and a radius half that of the concentric circle where the sampling point is located. To reduce noise, each sampling point undergoes Gaussian blurring, and the radius of the field represents the standard deviation of the Gaussian blur. Figure 2 illustrates the sampling mode utilized by FREAK. The receptive fields of the sampling points have overlapping areas, and Gaussian kernel functions of varying sizes are used to smooth the sampling points based on their distances from the features. Receptive fields of different sizes in the human retina exhibit comparable modes of action. By using overlapping receptive fields, more information can be acquired, resulting in a final descriptor that is more distinctive and easily distinguishable in features. Let *F* denote the FREAK descriptor, which is calculated as shown in Eq. (2).

2 $$\begin{aligned} F=\sum \limits _{0 \le a \le N} 2^{a} T\left( P_{a}\right) \end{aligned}$$

where $P_{a}$ is the ath sample point pair, and *N* is the length of the descriptor. $T\left( P_{a}\right)$ represents the comparison result between sample point pairs, and $T\left( P_{a}\right)$ is calculated, as shown in Eq. (3).

3 $$\begin{aligned} T\left( P_{a}\right) =\left\{ \begin{array}{l} 1, I\left( P_{a}^{r_{1}}\right) -I\left( P_{a}^{r_{2}}\right) >0 \\ 0, I\left( P_{a}^{r_{1}}\right) -I\left( P_{a}^{r_{2}}\right) \le 0 \end{array}\right. \end{aligned}$$

where $I\left( P_{a}^{r_{1}}\right)$ and $I\left( P_{a}^{r_{2}}\right)$ are the pixel values of the pairs of points after Gaussian blurring, and $P_{a}^{r_{1}}$ and $P_{a}^{r_{2}}$ are the coordinates of the pairs of points in the image. Under this sampling pattern, 43 sampling points are generated, resulting in 903 sampling point pairs. However, not all point pairs affect feature description and may even introduce redundancy. Therefore, the following steps are used to select the point pairs for dimensionality reduction. First, a matrix containing all feature descriptions is constructed, where each row represents the encoding of all the sampling point pairs for that feature. The mean of each matrix column is calculated. The columns of the matrix are then reordered from smallest to largest according to the distance between their variance and 0.5. The top 512 columns are then selected for the final binary description.

Finally, to achieve rotational invariance, FREAK adds gradient values to the descriptors to represent directional features.

###  Feature optimization

After feature extraction and description, the features must be further optimized by feature matching and mismatch removal.

First, the Hamming distance between the feature descriptions is calculated, and the initial coarse matching is completed by finding the feature with the smallest distance to another feature set through brute-force matching.

Second, the GMS algorithm divides the image into feature neighborhoods by creating grids, whereby correct matches are distinguished from mismatches by assuming that correct matches have more matching pairs that conform to the matching relationship in the neighborhood. This is based on the motion smoothness assumption. Thus, the number of matching pairs that conform to the matching relationship in the neighborhood is counted to determine the correct and incorrect matches, prior to fine matching.

Finally, to further reduce the number of false matches, the RANSAC method is used to eliminate false matches between images after obtaining the fine matching pairs from GMS. Specifically, four features are randomly selected from the matched features, and the parameter model that matches the maximum number of features is determined to be optimal through calculations and continuous iterations.

After coarse and fine matching and elimination of false matches, the remaining features are considered valid.

**Fig. 3:**
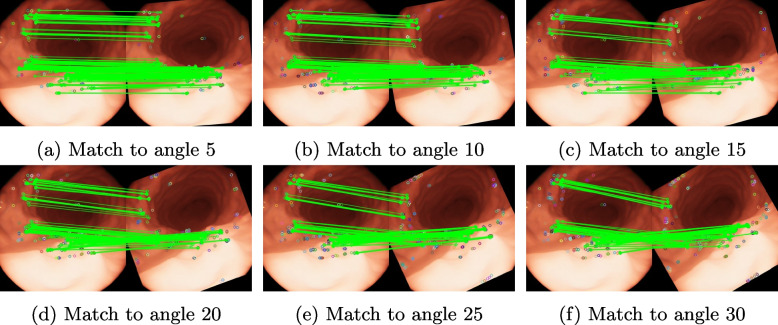
Performance under rotation frames

## Results and discussion

### Dataset

The simulation data were acquired in the VR-Caps platform designed by İncetan et al. [32]. This platform was built using Unity. VR-Caps generates a model of stomach organs based on CT images. Subsequently, stomach textures are created from the dataset acquired from patients and projected onto the model. The platform contains a virtual capsule endoscope that simulates a real capsule endoscopy procedure for filming. In this study, $320 \times 320$ stomach images were acquired using VR-Caps.

### Metrics and experimental details

The experiments were compared based on the correct number of features, matching scores, and running time.

The number of correct features was obtained by the GMS method, and false matches were eliminated using the RANSAC method. The matching score *MS* was calculated using Eq. (4).

4 $$\begin{aligned} M S=\frac{C M}{N} \end{aligned}$$

where *CM* is the number of correct features; and *N* is the number of features. *N* was calculated using Eq. (5).

5 $$\begin{aligned} N=\min \left( n_{1}, n_{2}\right) \end{aligned}$$

where $n_{1}$ is the number of extracted features for image 1; and $n_{2}$ is the number of extracted features for image 2. *N* is the smaller of the $n_{1}$ and $n_{2}$ values.

Correct features represent the number of feature recurrences when the image changes. The matching score which is the percentage of correct features among all features, indicates the efficiency of feature extraction.

In this study, the stomach images were randomly selected from the dataset simulated for the experiments. As there was a lack of real feature correspondence, the images were rotated during the experiments, to verify the effectiveness of the method in this study. The rotation angle ranged from $5^{\circ }$ to $30^{\circ }$ with a rotation step of $5^{\circ }$, which produced a total of six images. The original images were matched with the rotated images for features, and the rotation matrix was calculated according to the matching results and compared with the real data to estimate the rotation error.

In the image-supported endoscopic navigation system, some common methods can only obtain a small number of reliable features from multiple frames of endoscopic images. Thus, the number of feature matches between the first and subsequent frames gradually decreases as the view of the capsule endoscope changes over time. Therefore, stable tracking of features is a challenge in this application when the capsule endoscope view changes. In this study, one frame was extracted every 0.03 s in the simulation dataset.

### Experiment

In the experiments, maintaining rotational invariance by the proposed method was first verified using previously generated artificial images. The original images were then matched with the rotated images to verify the probability of feature repetition. The true rotation angles of the images were obtained because the test images were generated artificially. In this experiment, the homography matrix was calculated through feature matching and compared with the true angle to determine the error. The experimental results are listed in Table 1. Features extracted by the proposed method appear repeatedly during image rotation. As the rotation angle gradually increases, the feature extraction error increases, and the number of effective features decreases. In the results, when the rotation angle is $< 5^{\circ }$, the proposed method achieves high scores in all indicators. When the rotation angle is $< 20^{\circ }$, 40% of the features can be repeated. As the rotation angle increases, stability in feature extraction ability significantly decreases. The results are presented in Fig. 3.

**Fig. 4:**
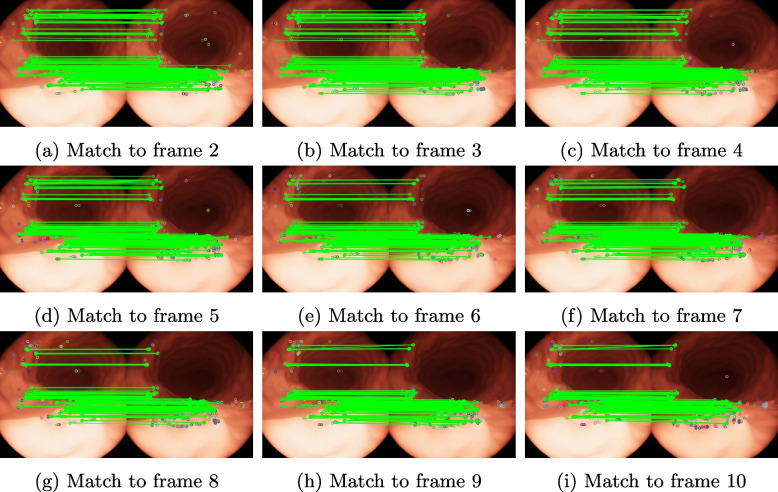
Performance under subsequent frames

Next, in the simulated environment of the virtual stomach, the rotational and translational motion states of the capsule endoscope inside the human body during examination were simulated. By matching the first frame with subsequent frames, the ability of the proposed method to stably track features was verified as the viewing angle of the capsule endoscope changed. The results are presented in Table 2, and the performance is displayed in Fig. 4.

According to the results, the number of matched features between the first and subsequent frames gradually decreases, indicating that the number of repeatable features also decreases. The proposed method can stably track nine consecutive frames, and the repeatability of the extracted features is over 50% for all nine consecutive frames. Except for possible errors when matching the sixth frame, the data exhibit fluctuations.

Based on the results of rotation and consecutive frame experiments, the proposed method works normally when the image undergoes small rotations and translations. The capsule endoscope is controlled by a doctor in the human stomach and does not undergo significant changes in the viewing angle; therefore, our method is applicable to capsule endoscopy.

In terms of time complexity, the Gaussian pyramid constructed using this method has eight levels, and the time complexity of the Gaussian smoothing operations is approximately *O*(1). Assuming that the sum of the pixels is *N*, the calculation complexity of the first-level image is *O*(*N*), and subsequent images are created by 1.5 times downsampling. Therefore, the total complexity of pyramid construction is *O*(*N*). Subsequently, feature extraction is performed for each image. By traversing all the pixels in the image, the proposed threshold calculation method requires iterating over each pixel in the neighborhood to compute the sum while finding the maximum and minimum values. Assuming *k* is the sum of the pixel neighborhood, this process has a time complexity of *O*(*k*). Further assuming that the number of pixels is *M*, the time complexity reaches ${O(M*k)}$. In terms of space complexity, the feature descriptor used in this study is consistent with that of the FREAK algorithm.

In feature optimization, the time complexity of a match based on the Hamming distance is *O*(*L*), where *L* represents the length of the feature description. Finally, the time complexity of the GMS algorithm is *O*(*A*), with *A* representing the number of features to match.

**Table 1:** Result under rotation scene

Rotation angle (degree)	Error	Correct feature	Matching score
5	0.019	280	0.743
10	0.045	240	0.692
15	0.054	142	0.432
20	0.072	143	0.433
25	0.089	133	0.367
30	0.111	134	0.325

**Table 2:** Result under simulated motion scenes

Index of frame matched to frame 1	Correct feature	Matching score
2	340	0.825
3	345	0.837
4	327	0.794
5	286	0.694
6	269	0.653
7	299	0.726
8	280	0.680
9	279	0.677
10	263	0.638

### Ablation experiments

First, the original feature-extraction method was compared with the adaptive feature-extraction method in two scenes. The original method extracted features using the fixed-threshold FAST method and described them using FREAK. The results demonstrate the improvements obtained by the proposed method. The results of the rotational scenes are listed in Tables 3 and 4 and those of the simulated motion scenes are listed in Tables 5 and 6.

**Table 3:** Correct features for rotation scene - comparison with the original method

Rotation angle (degree)	Original method	Proposed
5	0	280
10	0	240
15	0	142
20	0	143
25	0	133
30	0	134

**Table 4:** Matching score for rotation scene - comparison with the original method

Rotation angle (degree)	Original method	Proposed
5	0	0.743
10	0	0.692
15	0	0.432
20	0	0.433
25	0	0.367
30	0	0.325

**Table 5:** Correct features under simulated motion scenes - comparison with the original method

Index of frame matched to frame 1	Original method	Proposed
2	0	340
3	0	345
4	0	327
5	0	286
6	0	269
7	0	299
8	0	280
9	0	279
10	0	263

**Table 6:** Matching score under simulated motion scenes - comparison with original method

Index of frame matched to frame 1	Original method	Proposed
2	0	0.825
3	0	0.837
4	0	0.794
5	0	0.694
6	0	0.653
7	0	0.726
8	0	0.680
9	0	0.677
10	0	0.638

According to the results, the proposed method for feature extraction is significantly more powerful than the original method in extracting highly reproducible features for the same descriptor. As expected, the proposed method computes the corresponding threshold for all pixels in the feature-extraction range, demonstrating the adaptive nature of the approach.

The features extracted by the proposed method were compared against those obtained by different descriptors, such as SIFT, Brief, and SURF. The experiments were conducted under rotational and simulated motion scenes. The results of the rotational scenes are presented in Tables 7 and 8. The results for the simulated motion scenes are presented in Tables 9 and 10.

**Table 7:** Correct features under rotation scenes - comparison of different descriptors

Rotation angle (degree)	BRISK	SIFT	rBrief	Proposed
5	222	0	245	280
10	181	0	212	240
15	120	0	131	142
20	150	0	76	143
25	63	0	42	133
30	109	0	40	134

**Table 8:** Matching score under rotation scenes - comparison of different descriptors

Rotation angle (degree)	BRISK	SIFT	rBrief	Proposed
5	0.540	0.000	0.666	0.743
10	0.484	0.000	0.629	0.692
15	0.318	0.000	0.411	0.432
20	0.375	0.000	0.248	0.433
25	0.148	0.000	0.133	0.367
30	0.255	0.000	0.114	0.325

**Table 9:** Correct features under simulated motion scenes - comparison of different descriptors

Index of frame matched to frame 1	BRISK	SIFT	rBrief	Proposed
2	286	0	305	340
3	264	0	293	345
4	264	0	281	327
5	204	0	250	286
6	186	0	216	269
7	228	0	255	299
8	193	0	234	280
9	187	0	183	279
10	166	0	216	263

**Table 10:** Matching score under simulated motion scenes - comparison of different descriptors

Index of frame matched to frame 1	BRISK	SIFT	rBrief	Proposed
2	0.670	0.000	0.801	0.825
3	0.618	0.000	0.769	0.837
4	0.618	0.000	0.738	0.794
5	0.478	0.000	0.656	0.694
6	0.436	0.000	0.567	0.653
7	0.534	0.000	0.669	0.726
8	0.452	0.000	0.614	0.680
9	0.438	0.000	0.480	0.677
10	0.389	0.000	0.567	0.638

### Comparison experiments

In this experiment, several popular feature-extraction methods were compared with the method proposed herein to demonstrate the advantages of the proposed method.

**Fig. 5:**
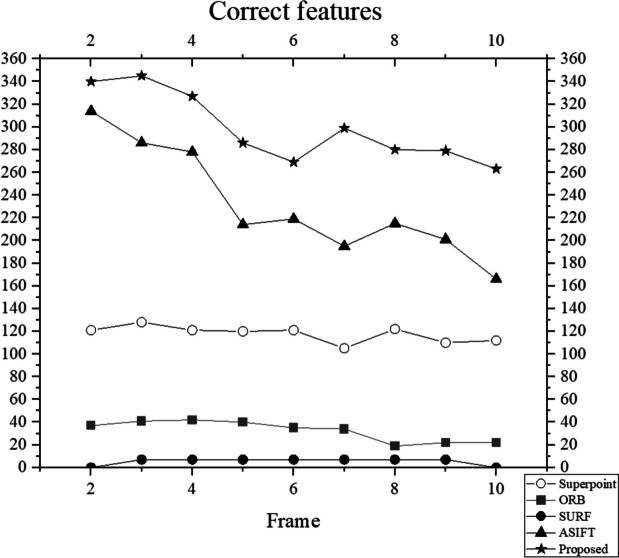
Correct features

**Fig. 6:**
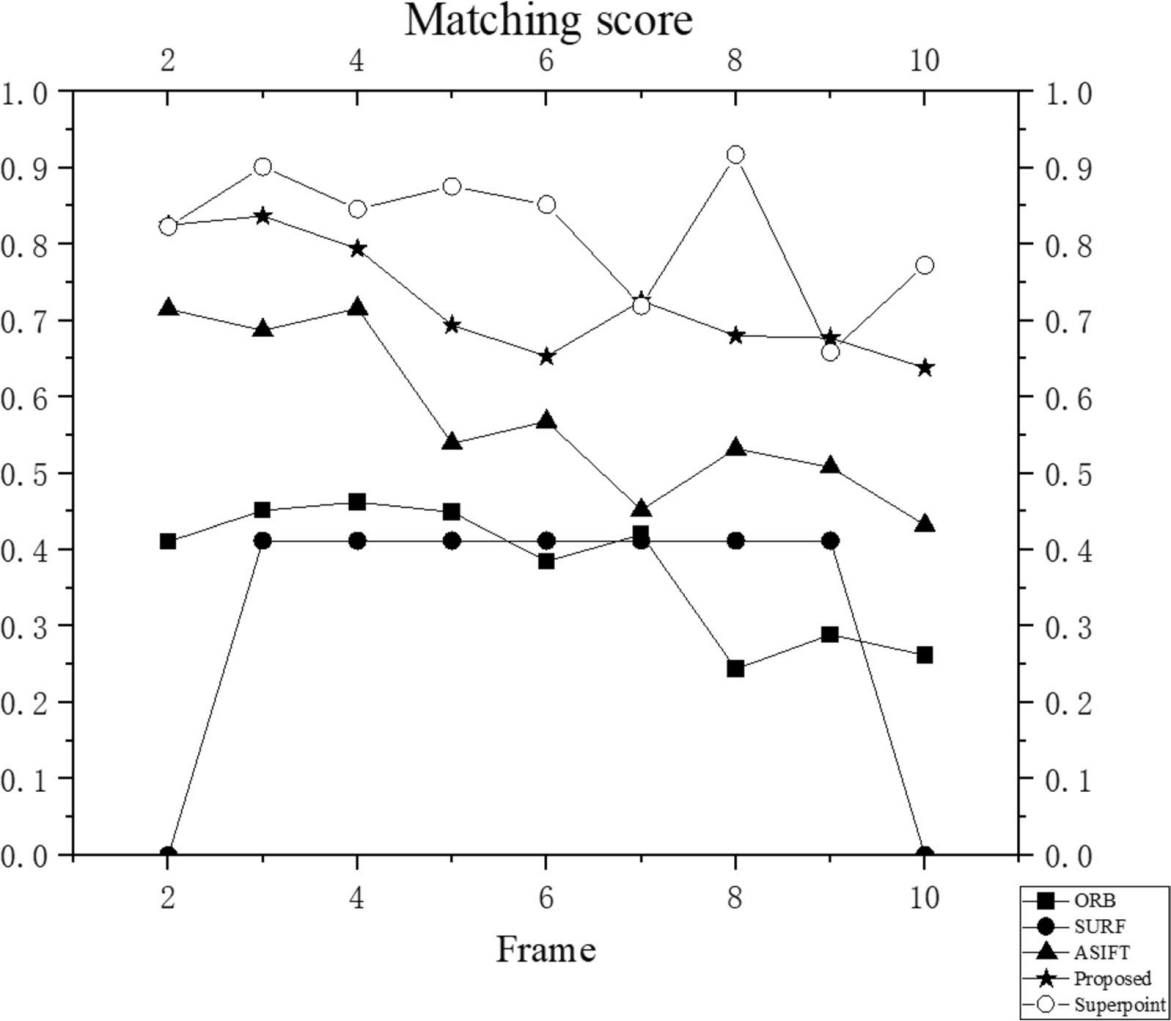
Macthing score

**Fig. 7:**
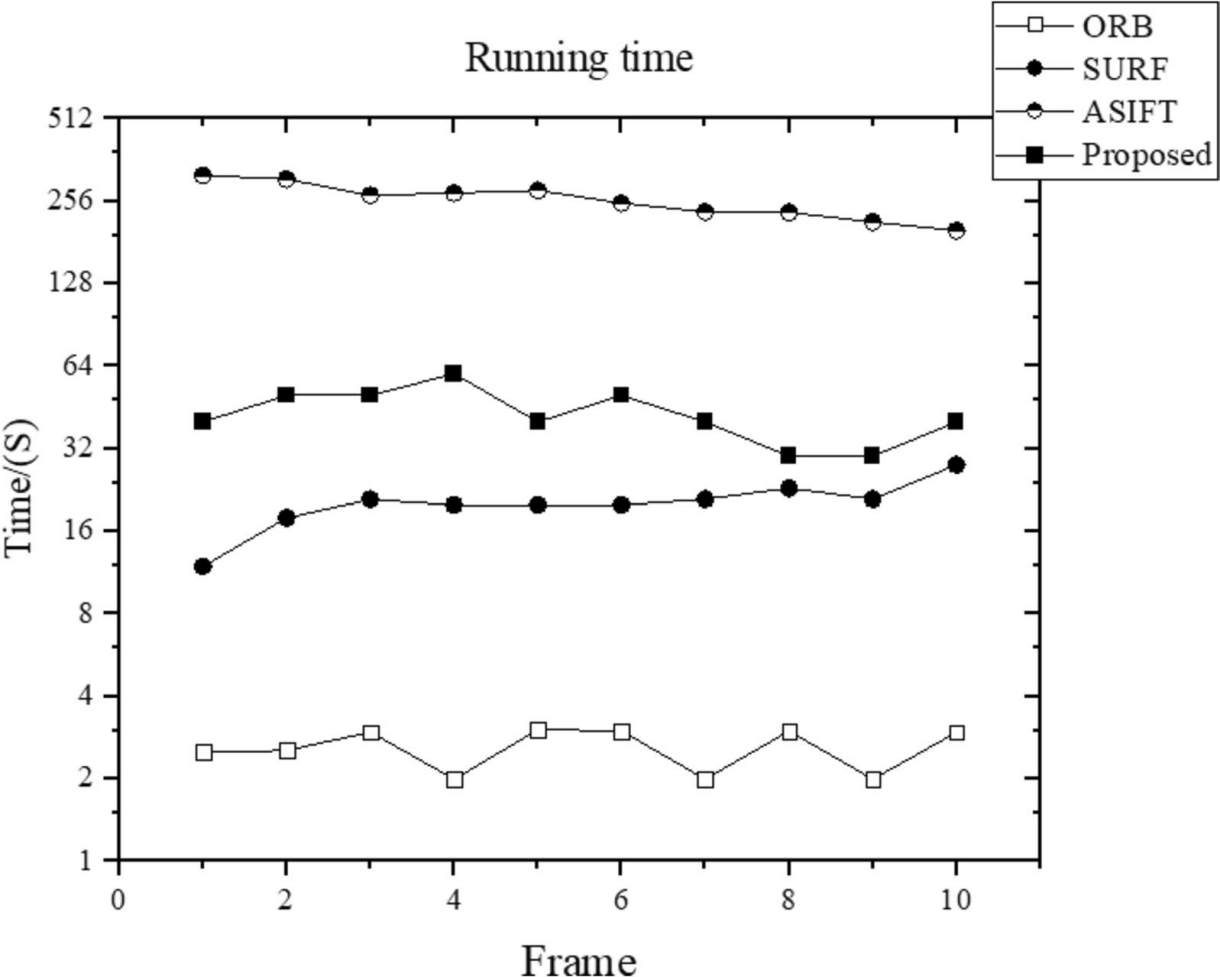
Running time

First, ORB was considered as a comparison method before improving it using the rBrief descriptor. Subsequently, SURF and ASIFT, which are improved SIFT methods, were considered. Recently, with the development of DL, numerous feature-extraction methods based on learning methods have been proposed. In the experiment, SuperPoint [25] was considered as the representative learning method. The number of correct features for the moving capsule endoscope were compared, as shown in Fig. 5. The matching-score comparison is shown in Fig. 6, and the running times are displayed in Fig. 7. It is worth noting that the experiments did not consider the running time of SuperPoint because this method converts the data into the style of Pytorch running on GPU, which is unfair to other methods.

The performance of SuperPoint, as a self-supervised DL model, depends on the quality and quantity of the training datasets. Capsule endoscopy datasets are rare because of the privacy protection of patients and hospitals, which affects the effectiveness of the model. Moreover, images of the human stomach may differ due to disease and individual body composition. The lack of a large dataset to support the portability of the model is another problem.

Additionally, the model structure of SuperPoint is complex and requires considerable computational resources and time for training and testing, which may not be suitable for application scenarios with limited resources.

The proposed method is used in medical application scenarios of capsule endoscopy, which require methods with reliable interpretability. Compared with the method used in this study, SuperPoint relies on a pre-trained feature extractor and does not have good interpretability. Although SuperPoint has the best performance in matching scores in Fig. 5, combined the Fig. 3, the method of this study can track at least 200 features, whereas the number of features tracked by SuperPoint is maintained at approximately 125. The number of features that appear repeatedly in adjacent frames is significantly higher. Medical aid with computer vision requires more valid support features. The method proposed in this study is a reliable traditional method that does not require GPU resources for training and testing and can extract the largest number of effective features. Considering resource efficiency and cost, the proposed method is a better choice for specific cases.

Moreover, compared with ASIFT, the average matching score of this method is improved by 5%, and the running time is 4/5 that of the ASIFT method.

### Verification of universality

**Fig. 8:**
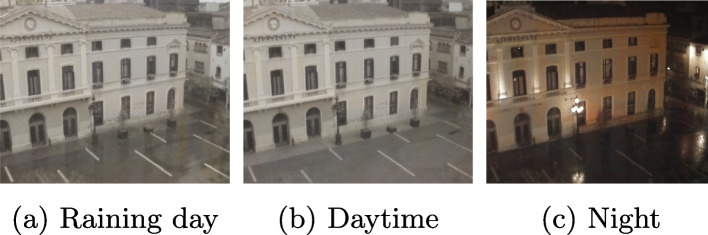
Images for verification

To verify that the method in this study is also applicable to other images, feature extraction was performed on images other than capsule endoscopes. This section describes the selection of images from Homography patches. The images used in the universality verification included rainy days, daytime, and nighttime, as shown in Fig. 8. The results of the number of correct features are shown in Table 11, and the matching score results are shown in Table 12. According to the experimental results, the traditional methods, SURF and ORB, do not work normally at night; however, the method in this study can obtain the most accurate features at night. Thus, the proposed AFFCEI works well when the illumination is changed to match that of natural scenes.

**Table 11:** Correct features

Match with daytime	SURF	ORB	ASIFT	AFFCEI
Rain	413	613	6208	4360
Night	0	0	469	523

**Table 12:** Matching score

Match with daytime	SURF	ORB	ASIFT	AFFCEI
Rain	0.279	0.294	0.500	0.443
Night	0	0	0.056	0.066

## Conclusions

This study proposes an improved feature extraction algorithm called AFFCEI for capsule endoscopic stomach images characterized by weak texture and uneven illumination. The algorithm uses an improved FAST feature-extraction method in the localization stage to extract a sufficient number of features. The threshold value of each pixel point is determined by calculating the gray-level contrast in the local neighborhood of the image, which is regionally adaptive and helps extract features more reliably even when the illumination changes. In the description stage, the features of the stomach image were accurately differentiated using the FREAK descriptor. The proposed method can be applied to extract features from images with poor texture. Overall, this approach significantly enhances feature extraction from capsule endoscopic stomach images and those of similar scenes.

## Data Availability

The datasets analyzed in the current study are available from the Virtual Capsule Endoscopy repository, https://data.mendeley.com/datasets/cd2rtzm23r/1.

## Abbreviations

ORB: Oriented FAST and rotated BRIEF
GMS: Grid-based motion statistics
AFFCEI: Adaptive threshold FAST and FREAK in capsule endoscopy image
RANSAC: Random sample consensus
SIFT: Scale-invariant feature transform
SURF: Speed up robust features
FAST: Feature from accelerated segment test
IC: Indigo carmine
rBRIEF: Rotation-aware BRIEF
DL: Deep learning
